# Expression of vascular endothelial growth factor receptor-2, epidermal growth factor receptor, cyclooxygenase-2, survivin, E-cadherin and Ki-67 in canine nasal carcinomas and sarcomas – a pilot study

**DOI:** 10.3389/fvets.2024.1388493

**Published:** 2024-08-29

**Authors:** Ljuba Anna Maria Pauly, Johannes Junginger, Gerhard Ulrich Oechtering, Marion Hewicker-Trautwein, Sarah Rösch

**Affiliations:** ^1^Department of Pathology, University of Veterinary Medicine Hannover, Foundation, Hannover, Germany; ^2^Small Animal Department, Ear, Nose and Throat Unit, Faculty of Veterinary Medicine, Leipzig University, Leipzig, Germany

**Keywords:** dogs, nasal tumors, chronic nasal discharge, T-categories, computed tomography, immunohistochemistry, tyrosine kinase inhibitors, cyclooxygenase inhibitors

## Abstract

**Background:**

Malignant (intra-) nasal tumors (NTs) are the most common cause of chronic nasal discharge in dogs. Besides radiation therapy, palliative therapy is necessary in some dogs. Therefore, studies on receptor expression have supported the utility of tyrosine kinase inhibitors (TKI) in dogs with nasal carcinomas. However, studies on receptor expression in nasal sarcomas are lacking.

**Materials and methods:**

This study evaluated the expression of vascular endothelial growth factor receptor-2 (VEGFR-2), epidermal growth factor receptor (EGFR), cyclooxigenase-2 (COX-2), Ki-67, survivin and E-cadherin in nasal carcinomas and sarcomas and compared it with tumor (T) categories based on computed tomography (CT).

**Results:**

In 26 dogs with NTs, cross sectional imaging and upper airway endoscopy with guided biopsy collection were performed, followed by histopathological examination of NTs, revealing 19 epithelial and 7 mesenchymal tumors. While EGFR and E-cadherin were only expressed by carcinomas, the following markers were expressed by both carcinomas and sarcomas without significant differences between tumor types and T-categories: VEGFR-2 (carcinomas and sarcomas 100%), COX-2 (carcinomas 63%, sarcomas 57%), survivin (carcinomas 100%, sarcomas 86%) and Ki-67 (median expression of 28.5% in carcinomas and 17.3% in sarcomas).

**Conclusion:**

Based on similarities in marker expression between canine carcinomas and sarcomas, clinical studies should further elucidate the use of TKI or COX-2 inhibitors as additional therapy in dogs with nasal sarcomas.

## Introduction

Canine nasal cavity tumors have a prevalence of 0.3–2.4% in dogs (1–3). However, they account for up to 10% of all canine tumors seen in oncology referral clinics (1–3). Nasal tumors are the most common cause of chronic nasal discharge in dogs (4–8). Most of nasal tumors are histologically malignant with nasal carcinomas more often observed than nasal sarcomas (6, 9). Up to date, radiation therapy is supposed to be the gold standard of therapy with median survival times (MST) of 8–19.7 months depending on the tumor size at time of diagnosis (10, 11).

Toceranib is an available tyrosine kinase inhibitor (TKI) that selectively inhibits the tyrosine kinase activity of VEGFR-2, platelet-derived growth factor receptor and the stem cell factor receptor KIT. It has been shown to result in clinical improvement in dogs with nasal carcinomas (12). Besides toceranib, selective COX-2 inhibitors as, e.g., firocoxib were used in dogs with nasal carcinomas and were shown to significantly improve quality of life in combination with radiotherapy (13). Newer adjuvant therapies could include monoclonal antibodies against EGFR or survivin inhibitors as survivin is an inhibitor of apoptosis, upregulated in various tumors and a potential target of novel antineoplastics (14, 15).

So far, studies in dogs with nasal sarcomas on receptor expression and clinical outcome of treatment with these drugs are lacking. Therefore, the aim of this work was the immunohistochemical characterization of EGFR, VEGFR-2, COX-2, Ki-67, survivin and E-cadherin in canine nasal sarcomas compared to carcinomas and to compare the expression to tumor size (T-categories). This was to test the hypothesis of significant differences in receptor expression between carcinomas and sarcomas, as well as between T-categories.

## Methods

### Selection criteria for animals

Medical records of dogs presenting to the Ear, Nose and Throat (ENT) Unit of the Small Animal Department of Leipzig University due to nasal discharge and the diagnosis of malignant nasal neoplasia between January 2015 and December 2017 were included and patient records retrospectively reviewed. Data analysis included patient demographics (age, breed, sex, clinical signs, therapy protocol, additional therapy, response, side effects, endpoints), results from the cross-sectional imaging, endoscopy as well as the result of the histopathological examination. Due to the low number of dogs, malignant tumors other than carcinomas and sarcomas were excluded.

Biopsies of the tumors were retrospectively used for immunohistochemistry. Nasal biopsies of healthy dogs without evidence of nasal discharge or nasal pathologies which were obtained during another approved prospective clinical trial (control dogs, see this separate section in methods) served as controls for immunohistochemical markers (16).

### Computed tomography and magnetic resonance imaging

A six-line spiral CT was used as described before (Philips Brilliance CT MX 8000 IDT 6, Philips Healthcare, Hamburg, Germany) (6). In one dog, cross sectional imaging had to be performed with a 3 T magnetic resonance imaging (MRI) scanner (Ingenia 3.0 T, Philips Healthcare, Hamburg, Germany). For helical CT scans, dogs were positioned in sternal recumbency with the hard palate fixed parallel to the table with a conventional positioning restrainer. Nonionic contrast medium (Ioversol, Optiray 300 mg/mL, Covidien S.p.A., Segrate, Milan, Italy) was used to distinguish contrast-enhanced tissues from nasal discharge. Images were examined for a (1) nasal mass (size), (2) bone lysis, (3) effects on the septum nasi, (4) effects on the frontal sinus, (5) presence of metastases in the lung or lymph nodes. Heterogenous contrast enhancement in lymph nodes and enlargement of lymph nodes were characteristics suspicious for malignancy (17). According to Adams et al. (18), dogs were staged in four T-categories (Table 1).

**Table 1:** Adams’ proposed anatomical staging system for canine nasal tumors (18); T = T-category.

Stage	Tumor characteristics	Median overall survival (months) after radiation therapy
T1	Confined to one nasal passage or frontal sinus, with no bony involvement beyond turbinates	23.1
T2	Any bony involvement (beyond turbinates), without evidence of orbit, subcutaneous, or submucosal mass	14
T3	Orbit or nasopharynx involved, or a subcutaneous, or submucosal mass	15.7
T4	Tumor causing destruction/lysis of the cibriform plate	6.7

### Control group and ethics approval

Nasal biopsies of healthy dogs were re-used from a prospective clinical trial based on an animal experiment application with ethics review and approval (Regional Council of the Free State of Saxony, Leipzig, Germany: TVV Animal experiment subject 02/18) (16). All examinations were performed in a standardized manner and in accordance with the guidelines and regulations according to TVV 02/18. In this cited study, dogs were proven to be healthy by the results of clinical examination, whole-body CT, endoscopy of the upper airways with negative bacteriological and mycological examinations of nasal mucosal swabs and histopathological examination of nasal mucosal biopsies (16). Histopathologic examination of nasal mucosal biopsies was unremarkable. Clinical control examinations of the dogs 3 months after intervention revealed no abnormalities (16).

### Histopathological examination

After taking biopsy samples of the NTs under endoscopic visualization and guidance, the samples were fixed in 10% formalin and embedded in paraffin. The initial histopathological examination was performed on hematoxylin-eosin (H&E) sections by Antech Lab Germany GmbH, Tierpathologie Munich, Germany with Dr. W. von Bomhard, Dipl. ECVP. Histopathological diagnosis was reevaluated and confirmed by a Dipl. ECVP (MHT).

### Immunohistochemistry and evaluation

For immunohistochemistry, 3 μm thick sections were applied on Superfrost® Plus slides. The avidin-biotin-complex (ABC) method was used according to routine protocols (19). Antigen retrieval was achieved by heat induction with citrate buffer or, for EGFR, with pronase E. For EGFR and COX-2, the detection was amplified by the use of biotinylated tyramine followed by a second application of ABC (20). Sections were stained with 3,3′-diaminobenzidine and counterstained with Mayer’s haemalum solution. For negative controls, the specific primary antibody was replaced by a normal serum or the corresponding isotype antibody of the same species. A list of the antibodies used in this study, their sources of supply and references for use on dog tissues is to be found in Table 2.

**Table 2:** Antibodies used for immunohistochemical detection of target antigens and their references.

Antibody	Source of supply	Clone	Isotype	Dilution	Pretreatment	Incubation	Cross-reactivity with equivalent proteins of canine origin (supplier’s information)	References that used the same clone for immunohistochemistry in canine tissue
EGFR	BioPrime (CAT: EG105)	111.6	mAb, mouse IgG1	1:400	Pronase E, 37°C	75 min., RT	no details	Shiomitsu et al. (21), Hocker et al. (22), and Sabattini et al. (23)
VEGFR-2	Santa Cruz Biotechnology (Flk-1, CAT: sc6251)	A-3	mAb, mouse IgG1 ƙ	1:50	MW	overnight at 4°C	no details	Wolfesberger et al. (24), Diessler et al. (25)
COX-2	Santa Cruz Biotechnology (C-20, CAT: sc-1745)	-	pAb, goat IgG	1:400	MW	75 min., RT	yes	Millanta et al. (26), Impellizeri and Esplin (27)
Ki-67	Dako (CAT: M7240)	MIB-1	mAb, mouse IgG1 ƙ	1:100	MW	75 min., RT	yes	Scase et al. (28), Thompson et al. (29), Ciaputa et al. (30), Fu et al. (31), Sokołowska et al. (32), Rodrigues et al. (33)
Survivin	NOVUSBio (CAT: MB500-201)	-	pAb, rabbit IgG	1:500	MW	75 min., RT	yes	Scase et al. (28), Rebhun et al. (34), Fu et al. (31)
E-cadherin	Biosciences BD Transduction Laboratories (CAT: 610181 and 610,182)	36/E-Cadherin	mAb, mouse IgG2a ƙ	1:100	MW	75 min., RT	yes	Aresu et al. (35)
Cyto-keratins	Dako (CAT: M3515)	AE1/AE3	mAb, mouse IgG1 ƙ	1:500	MW	75 min., RT	yes	Grieco et al. (36), Sako et al. (37), Matos et al. (38), Burgess et al. (39)
Vimentin	Dako (CAT: M0725)	V9	mAb, mouse IgG1 ƙ	1:100	none	75 min., RT	no details	Destexhe et al. (40), Koenig et al. (41), Grieco et al. (36), Burgess et al. (39)

EGFR: epidermal growth factor receptor, VEGFR-2: vascular endothelial growth factor receptor-2 = Flk-1 = fetal liver kinase-1, COX-2: Cyclooxigenase-2, RT: room temperature, CAT: catalog number, mAb: monoclonal antibody, pAb: polyclonal antibody, MW: microwave, IgG: Immunglobulin G.

Necrotic and inflammatory areas were omitted from the assessment. For a better comparability, the immunohistochemical staining was evaluated according to the methods described for each antibody in the literature (21, 31, 42–49) and therefore analyzed. Therefore, stainings for five antibodies, EGFR, VEGFR-2, COX-2, survivin and E-cadherin, were analyzed semiquantitatively.

**Figure 1:**
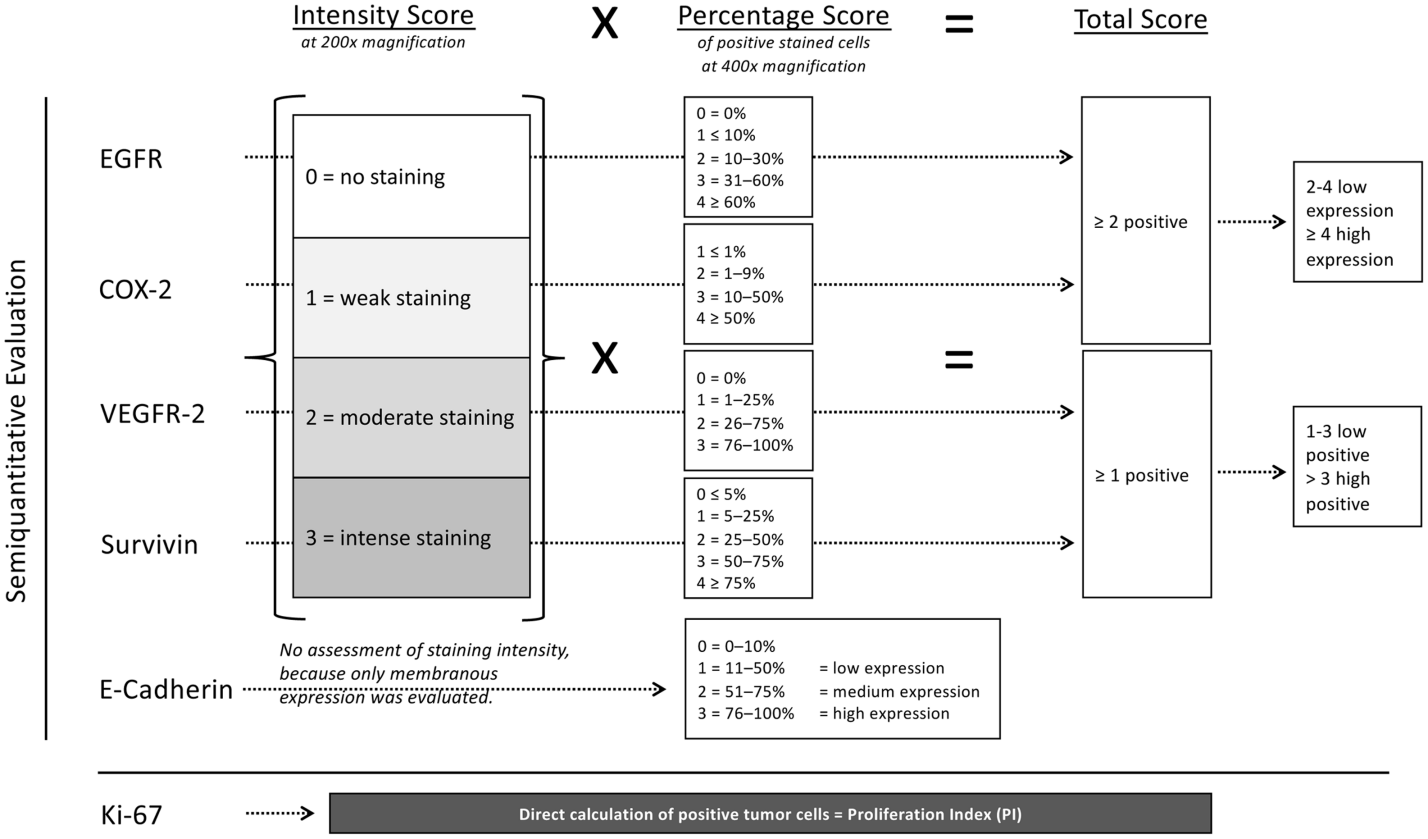
Illustration of the evaluation of immunohistochemical staining with the different antibodies based on published analysis methods. EGFR, COX-2, VEGFR-2, survivin and E-cadherin were evaluated semiquantitatively, in contrast to Ki-67, where the positive tumor cells were counted using image analysis software (Fiji Image J, Fiji contributors) on digital images taken by a photomicroscope. For EGFR, COX-2, VEGFR-2 and survivin, a total score (intensity score multiplied by the individual percentage score) was used. For E-cadherin, the percentage score was evaluated only. For Ki-67 the proliferation index was evaluated.

In four of five antibodies, EGFR, VEGFR-2, COX-2 and survivin, the total score (Figure 1) was calculated by multiplication of a determined intensity score (assessment at 200x magnification; 0 (no staining), 1 (weak staining), 2 (moderate staining) or 3 (intense staining)) with an individual percentage score (assessment at 400x magnification) in each antibody. The individual percentage score is summarized in Figure 1 and was calculated as follows.

For EGFR, the percentage score was classified as 0 = 0%, 1 = <10%, 2 = 10–30%, 3 = 31–60%, and 4= > 60% (21). If the total score was ≥2, the tumor was considered to be positive. The expression of VEGFR-2 was evaluated with a classification of percentage scores from 0 to 3 (0, 1–25%, 26–75% and 76–100%) (42). The tumor was considered to be VEGFR-2 positive if a total score of ≥1 was present. The COX-2 immunostaining was classified into percentage scores of 1–4 (1 = <1%, 2 = 1–9%, 3 = 10–50%, 4= > 50%) with a total score of ≥2 positive for COX-2 (43). The expression is classified as low if the total score is 2–4. Tumors with a total score ≥ 4 show a high COX-2 expression (44). For survivin, the percentage scores were classified in 0 = <5%, 1 = 5–25%, 2 = 25–50%, 3 = 50–75% and 4= > 75% with a total score of 0 evaluated as negative, 1–3 as low and > 3 as high survivin expression (31).

Because in the fifth’s antibody E-cadherin only membranous expression was evaluated semiquantitatively, staining intensity was not assessed and only the percentage score was determined. Therefore, E-cadherin expression was classified into percentage scores: 0 (negative) = 0–10%, 1 (low expression) = 11–50%, 2 (medium expression) = 51–75%, 3 (high expression) = 76–100% (45).

In contrast to these described five antibodies, for evaluating the immunostaining of Ki-67, images of at least seven randomized fields in areas of the most intense staining were taken at 400x magnification using a photomicroscope (Olympus: fluorescence microscope BX51, C-mount microscope camera adapter, U-CMAD3 and U-TV1X-2 and microscope camera, DP72). Using image analysis software,^1^ at least 500 tumor cells were then counted (46–49). The percentage of positive tumor cells was calculated and given as the proliferation index (PI). To classify Ki-67 expression into low versus high, the percentage median value of all tumors was used as the cut off value (28, 31, 47).

To further confirm the histopathological diagnosis, all samples were analyzed for the expression of cytokeratins and vimentin.

**Figure 2:**
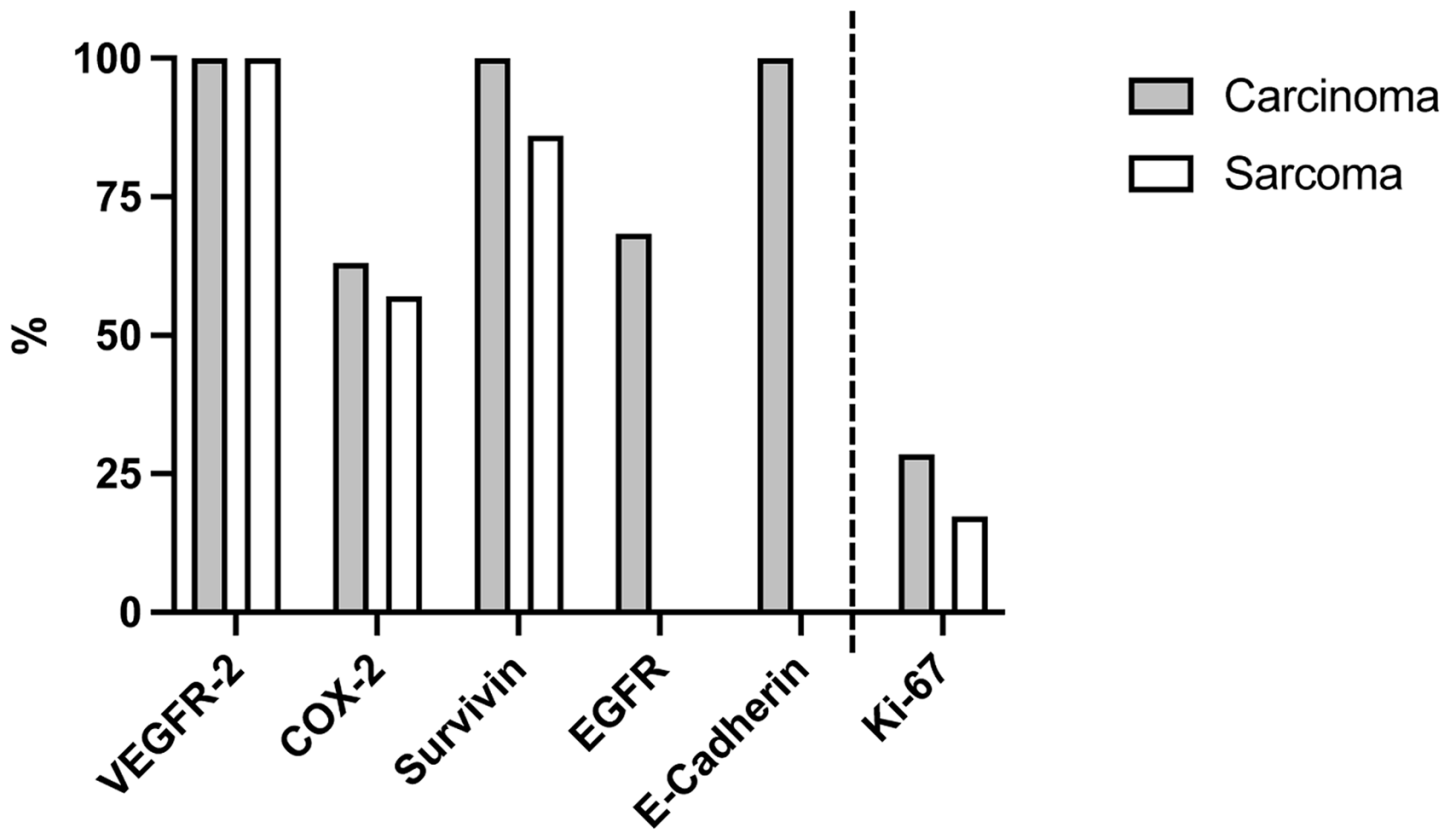
Expression of immunohistochemical markers in canine nasal carcinomas (*n* = 19) and sarcomas (*n* = 7). Carcinomas: 100% were positive for VEGFR-2, 63.2% for COX-2, 100% for survivin, 68.4% for EGFR and 100% for E-cadherin. Sarcomas: 100% were positive for VEGFR-2, 57.1% for COX-2, 85.7% for survivin and 0% for EGFR and E-Cadherin. The proportion of positive tumor cell nuclei for Ki-67 of the total number of counted tumor cell nuclei (proliferation index, PI) was 28.5% of nasal carcinomas and 17.3% of sarcomas. Because of the different meaning of [%] in this context-Ki-67 was separated in the bar diagram by a dashed line.

**Figure 3:**
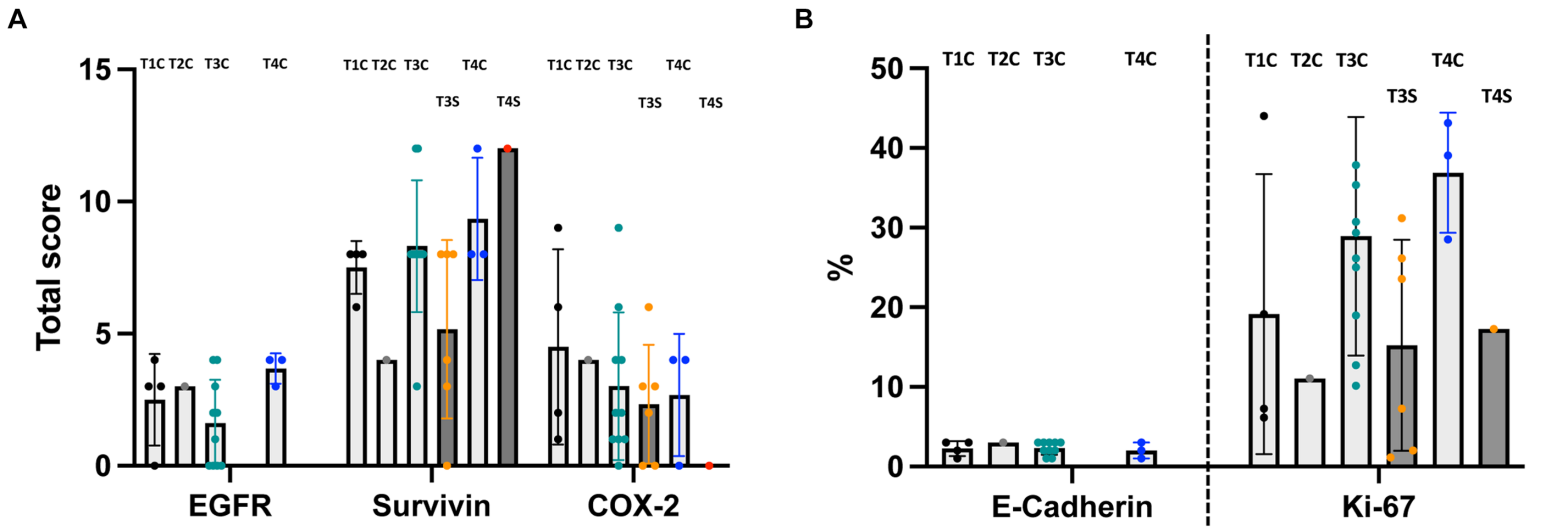
Comparison of the expression of immunohistochemical markers between canine nasal tumors of different T-categories (mean ± SD; order: T1, T2, T3, and T4 - with light gray bars representing carcinomas (C) and dark gray bars representing sarcomas (S)). **(A)** EGFR, survivin and COX-2. **(B)** E-Cadherin and Ki-67. Sarcomas are considered in **(A)** and **(B)** separately (dark gray bars with orange dots). There is no significant difference in tumor expression according to tumor size or tumor entity in the same T-category. **(B)** Because of the different meaning of [%] as PI in the context of Ki-67 - it was separated in the bar diagram by a dashed line.

The different calculation methods and the resulting total score in EGFR, COX-2, VEGFR-2 and survivin, in contrast to percentage score in E-cadherin and proliferation index in Ki-67, are the reason for the separation of data in the Figures 2, 3B by dashed lines.

### Statistics

Statistical analyses were performed using GraphPad Prism (v7 GraphPad Software, La Jolla, CA, United States). Data were tested for normality using the D’Agostino and Pearson Normality Test and the Shapiro–Wilk Normality Test. Normally distributed data were specified with mean ± SD, while non-parametric data were specified with median and interquartile range (IQR). Comparison among parametric data was made after testing for equality of variance by Brown-Forsythe test and Bartlett’s test, by One-way ANOVA or among non-parametric data by Kruskal-Wallis Test. For posthoc pairwise comparison of two parametric data, the unpaired T-test was used and in case of non-parametric data the Mann–Whitney test. A *p*-value <0.05 was considered significant.

## Results

### Clinical data

Twenty-six dogs with intranasal tumors were included. Median age was 10 years (IQR 9.1–13) and mean body weight was 23.4 kg (SD ± 11.1 kg). Six dogs were female (23%), 8 female-spayed (31%), 8 male (31%) and 4 male-castrated (15%).

### Tumor stages

Nasal tumors were detected in 50% in the left (13/26), in 46% in the right (12/26) and in 4% in both nasal cavities (1/26). Diagnosis was made by endoscopy (26/26) and CT (24/26) and/or MRI (1/26).

Of 25/26 dogs investigated by CT and/or MRI, 4 were grouped in T-category 1 (T1; all carcinomas), one in T-category 2 (T2; carcinoma), 16 dogs in T-category 3 (T3; 10 carcinomas (incl. one dog diagnosed with MRI) and 6 sarcomas) and 4 dogs in T-category 4 (T4; 3 carcinomas and one sarcoma). In one dog, no CT-or MRI-examination was performed due to financial restrictions of the owners, therefore a grouping into T-categories was not possible.

### Histopathological findings

19/26 nasal tumors were diagnosed as carcinomas (6 undifferentiated carcinomas, 6 adenocarcinomas, 5 transitional cell carcinomas und 2 squamous cell carcinomas) and 7/26 were diagnosed as sarcomas (3 chondrosarcomas, 2 osteosarcomas, one chondro- and osteoblastic sarcoma und 1 undifferentiated sarcoma).

### Clinical signs

**Figure 4:**
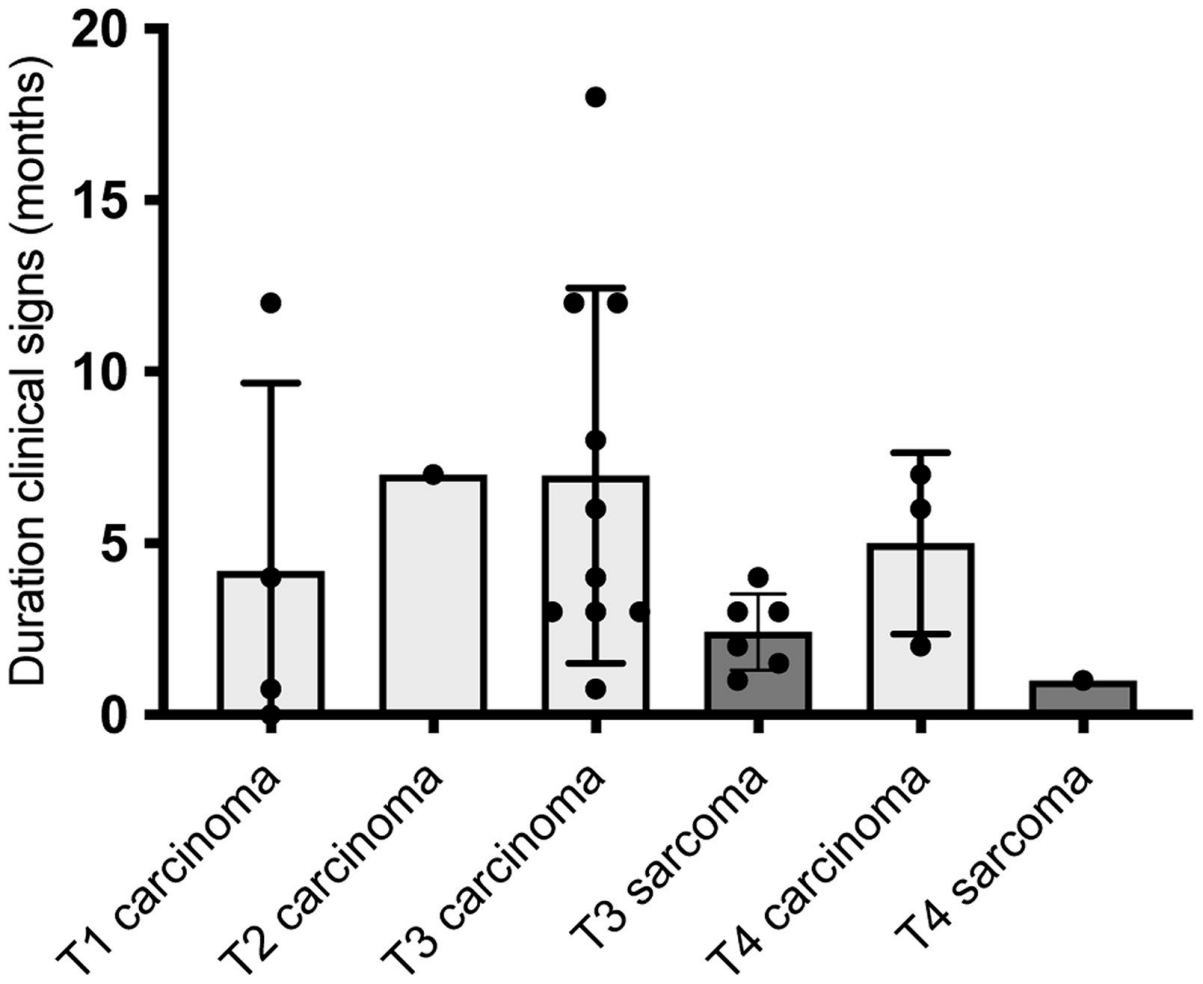
Duration of clinical signs in 25/26 dogs with nasal tumors in months in correlation to tumor size in cross sectional imaging (T = T-category; CT in 24/25 dogs, MRI in 1/25 dogs). Duration of clinical signs of the dogs is presented in bar diagrams with mean ± SD. Time was calculated by the owners from onset of clinical signs until the timepoint when diagnostics in anesthesia were performed at the ENT-Unit of the Small Animal Department of the Leipzig University due to nasal discharge. There was no significant difference of different T-categories in duration of clinical signs (Ordinary one-way ANOVA, *p* = 0.4350) or between carcinomas and sarcomas (Two tailed Mann–Whitney test, *p* = 0.0526). Therefore, tumor size at presentation was supposed not only to be correlated with duration of disease, but also with different speed of growths.

At presentation for further diagnostics, clinical signs as, e.g., nasal discharge (100% of the dogs) had been observed by the owner for a median duration of 3 months (IQR 2–6.3) (Figure 4). There was no statistically significant difference in the duration of clinical signs until diagnostics according to T-category (*p* = 0.9122), also when carcinomas were evaluated according to size separately from sarcomas (Figure 4). Duration of clinical signs in dogs with T3 sarcomas appeared numerically shorter than in dogs with T3 carcinomas, however, it was not significantly different (*p* = 0.0526).

### Immunohistochemical expression patterns in carcinomas versus sarcomas

The expression patterns of VEGFR-2, COX-2, Ki-67 and survivin were comparable between nasal carcinomas and sarcomas (Figure 2).

VEGFR-2 was positive in 100% of carcinomas and sarcomas. The median total expression score was 3 (IQR 0–9) in carcinoma and 3 (IQR 0–6) in sarcoma in a possible total score frame of 0–9.

63.2% (12/19) of carcinomas and 57.1% (4/7) of sarcomas showed positive COX-2 expression. In a score range of 0–12, the median total scores of COX-2 expression was 2 (IQR 1–4) in carcinomas and 2 (IQR 0–3) in sarcomas (*p* = 0.3135).

Survivin was expressed in 100% of carcinomas and in 85.7% of sarcomas (6/7). Both, carcinomas and sarcomas, had a median survivin expression with a total score of 8 (IQR nasal carcinomas 8–8 and sarcomas 3–8; *p* = 0.2107).

Median Ki-67 expression was 28.5% (IQR 12.7–37.9%) in carcinomas and 17.3% (IQR 2–26.2%) in sarcomas (*p* = 0.0566).

**Figure 5:**
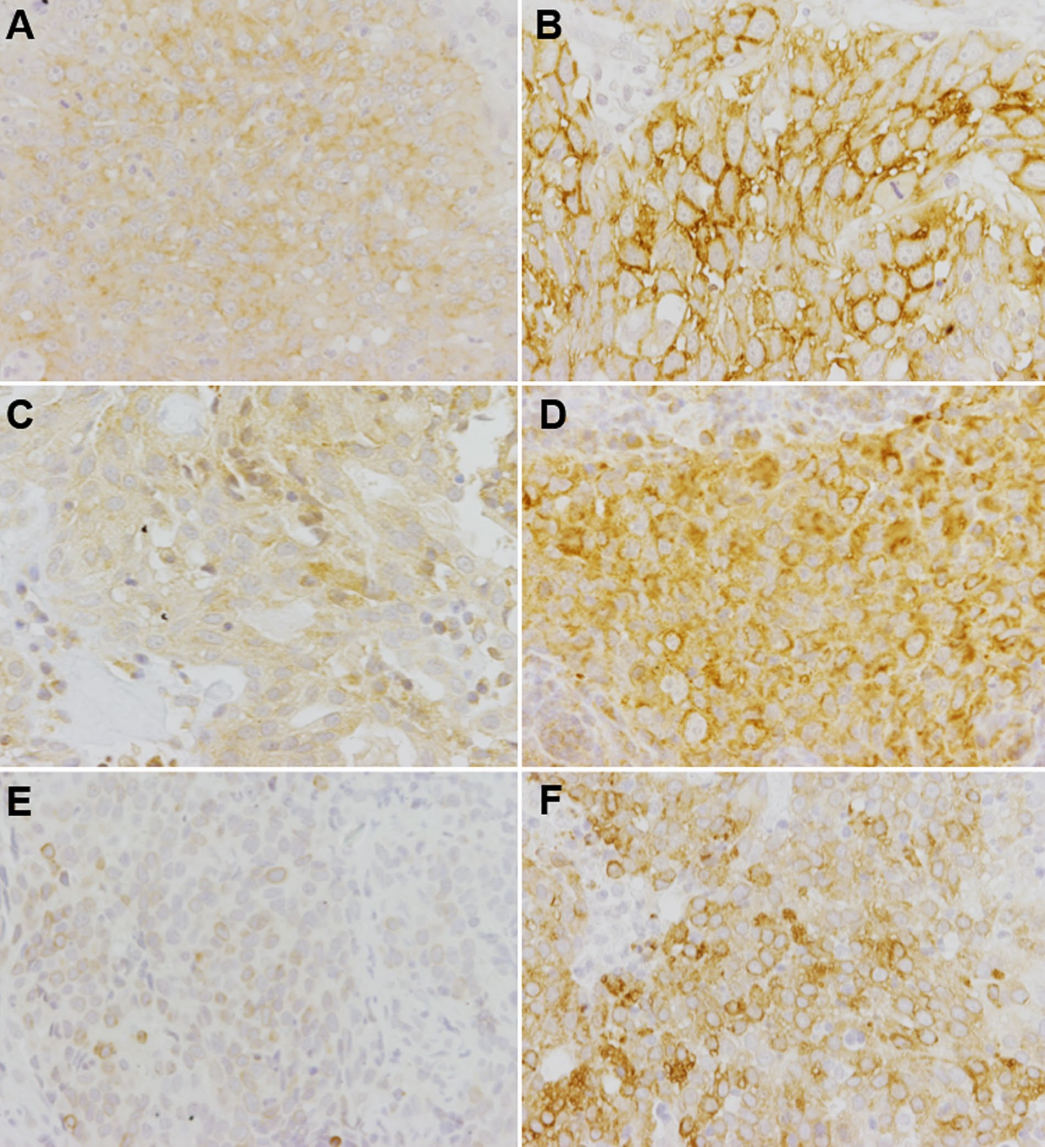
Examples of immunohistochemical stainings for the detection of EGFR **(A,B)**, VEGFR-2 **(C,D)** and COX-2 **(E,F)** in malignant canine nasal tumors. Representative pictures are displayed for low target protein expression **(A,C,E)** and high target protein expression **(B,D,F)**. Different histopathological tumor types are shown at a magnification of 200x. **(A)** Carcinoma with membranous-cytoplasmic staining pattern, total score 6. **(B)** Squamous cell carcinoma with membranous staining pattern, total score 12. **(C)** Transitional cell carcinoma with cytoplasmic staining pattern, total score 3. **(D)** Carcinoma with cytoplasmic staining pattern, total score 6. **(E)** Carcinoma with cytoplasmic perinuclear staining pattern, total score 4. **(F)** Carcinoma with cytoplasmic perinuclear staining pattern, total score 9.

EGFR and E-cadherin were expressed in carcinomas, but not in sarcomas. 68.4% (13/19) of nasal carcinoma were positive for EGFR with a median total score of 3 (IQR 0–9) in a possible total score frame of 0–12. 100% of the carcinomas were positive for E-cadherin with a median percentage score of 3 (IQR 2–3). Examples for the immunohistochemical staining patterns of EGFR, VEGFR-2 and COX-2 in this study are displayed in Figure 5.

### Immunohistochemical expression patterns did not differ between T-categories

The marker expression was compared in different categories based on tumor size (T-category; Adams et al. (18)). Twenty-five dogs with cross sectional imaging were included (carcinomas *n* = 18 and sarcomas *n* = 7). One dog with nasal carcinoma and without cross sectional imaging was excluded. Expression of the markers did not differ significantly between tumors in comparison of different T-categories, even if sarcomas (Figures 3A,B; orange points) were evaluated separately.

EGFR expression was not significantly different in the four T-categories of nasal carcinomas with a median total score of 3 (IQR 0.8–3.8) in T1 (*n* = 4), a total score of 3 in T2 (*n* = 1), a median total score of 1.5 (IQR 0–3.3) in T3 (*n* = 10) and a median total score of 4 (IQR 3–4) in T4 carcinomas (*n* = 3).

Evaluation of survivin expression revealed a median total score of 8 (IQR 6.5–8) in carcinomas in T1 (*n* = 4), a total score of 4 in one carcinoma in T2, a median total score of 8 (IQR 8–9) in carcinomas in T3 (*n* = 10), a median total score of 6 (IQR 2.5–8) in sarcomas in T3 (*n* = 6) and a median total score of 8 (IQR 8–12) in T4 carcinomas (*n* = 3), as well as 12 in T4 sarcoma (*n* = 1). Survivin expression was not significantly different in the four T-categories or between carcinomas and sarcomas.

For COX-2 expression, the median total score was 4 (IQR 1.25–8.25) in T1 carcinomas (*n* = 4), 4 in one T2 carcinoma, 2 (IQR 1–4.5) in T3 carcinomas (*n* = 10), 2.5 (IQR 0–3.8) in T3 sarcomas (*n* = 6), 4 (IQR 0–4) in T4 carcinomas (*n* = 3) and 0 in T4 sarcoma (n = 1). COX-2 expression was not significantly different in the four T-categories.

Ki-67 index was 13.2% (IQR 6.4–37.8%) in T1 (*n* = 4), 11.1% in T2 (*n* = 1), 27.8% (IQR 17.4–36%) in T3 carcinomas (*n* = 10) and 15.4% (IQR 1.8–27.4%) in T3 sarcoma (*n* = 6) and 39% (IQR 28.5–43.1%) T4 carcinomas (*n* = 3) and 17.3% T4 sarcoma (*n* = 1). The median values of Ki-67 expression did not differ significantly between the different T-categories.

100% of carcinomas expressed E-cadherin. The E-cadherin expression of the nasal carcinomas showed a median percentage score of 2.5 (IQR 1.3–3) in T1 (*n* = 4), 3 in T2 (*n* = 1), 2.5 (IQR 1.8–3) in T3 (*n* = 10) and 2 (IQR 1–3) in T4 (*n* = 3). E-cadherin expression was not significantly different between the four T-categories.

## Discussion

Nasal carcinomas represent two thirds of nasal cavity tumors in dogs (1, 3, 50–52). Consistent with literature, the majority of nasal cavity tumors in the present study were carcinomas (73%), whereas sarcomas were diagnosed in only 27% of dogs. Consequently, the number of investigated sarcomas (*n* = 7) in the present study is limited and a larger number of sarcomas would have been necessary to substantiate the results of this study. However, as, in contrast to canine nasal carcinomas, data about expression levels in canine nasal sarcomas are missing, the present study aimed to investigate certain markers in both canine nasal carcinomas and sarcomas by immunohistochemistry. Investigated markers included EGFR, VEGFR-2, COX-2, survivin, Ki-67 and E-cadherin. All examined tumor biopsies were obtained under endoscopic visualization and guidance and histopathological diagnosis was made by two pathologists. Comparable to other reports (3, 52, 53) undifferentiated carcinomas and adenocarcinomas were the most common type of carcinomas and chondrosarcomas were the most frequent type of sarcomas in our study. Immunohistochemistry showed that canine nasal carcinomas and sarcomas did not differ significant in expression of VEGFR-2, COX-2, survivin and Ki-67.

VEGFR-2 is a very important molecular target in anti-angiogenic tumor therapy, as it leads to endothelial cell proliferation and migration together with its ligand VEGF-A, and angiogenesis is a central factor in tumor growth (54). In carcinomas of the present study, VEGFR-2 expression was present in 100% (19/19) of the cases. This is in accordance with former studies that showed high expression of VEGFR-2 in canine nasal carcinomas (22, 42). In sarcomas of the present study, VEGFR-2 expression was similar to carcinomas. Therefore, TKIs as, e.g., toceranib, which inhibits VEGFR-2 with anti-angiogenic effects, may be therapeutically useful in sarcomas, as it is also described for carcinomas (12). In that study, toceranib already showed clinical benefit in 71% (5/7) of nasal tumor dogs in a study involving a total of 85 dogs with various solid tumors, 7 of which had nasal carcinomas (12). 1/7 (14%) dogs with nasal carcinoma experienced complete remission and 4/7 (57%) dogs had stable disease. 4/7 (57%) dogs received radiotherapy prior to the treatment with toceranib. It should be noted that only 2/7 dogs in that study were reassessed by CT after therapy with toceranib. In the remaining 5 dogs, improvement in clinical signs was considered a clinical benefit (12). There are two more recent publications about the use of toceranib in canine nasal carcinomas supporting the benefit of toceranib as adjuvant treatment (55, 56). One study demonstrated that toceranib in addition to radiotherapy improved the clinical benefit rate significantly compared to the group treated with radiotherapy alone (56). The other study showed a decrease of clinical signs in dogs with nasal carcinomas treated with toceranib alone (55).

In this study, the number of COX-2-positive carcinomas did not differ significantly from the number of COX-2-positive sarcomas. A similarly strong expression of COX-2 as in this study has already been demonstrated in earlier studies in nasal carcinomas and sarcomas in dogs (27, 43, 44, 57–59). However, both tumor types have never been analyzed and compared between each other. In the present study, there were no significant differences in COX-2 expression between the different T-categories, which is in accordance to a former study on canine nasal carcinomas by Fu (44). Immunohistochemical evaluation of COX-2 expression in other canine tumors does not seem to necessarily predict whether and how the tumor will respond to COX-2 inhibitors (60–63). It is possible that COX-2 inhibitors achieve their antineoplastic effect not exclusively via inhibition of COX-2, but also via COX-independent mechanisms (63). However, the COX-2 inhibitor firocoxib, given in addition to radiotherapy, already significantly improved the quality of life in dogs with nasal carcinomas compared to patients treated with radiotherapy alone (13).

In this investigation, 100% of carcinomas (19/19) and 86% of sarcomas (6/7) were survivin positive. This is in accordance with studies in which survivin expression was detected in 85% (28/33) to 100% (5/5) of canine nasal carcinomas (31, 64). In canine nasal carcinomas and human nasopharyngeal carcinomas, a significant correlation between a high survivin expression and a higher clinical stage as well as a shorter survival time has been demonstrated (44, 65, 66). Although there was a tendency for higher survivin expression in higher T-categories, the expression was not significantly different between T-categories or tumor entity in the present study.

For Ki-67, the median PI in the carcinoma group in the present study was 28.5%, which is consistent with the median Ki-67 expression of 28.5% in the study by Fu (44), in which only nasal canine carcinomas were investigated. In our study, sarcomas revealed a median PI of 17.3% which was not significantly different compared to carcinomas. Fu (44) demonstrated an association between a high Ki-67 expression and an advanced tumor stage (T3 and T4). However, this was not detected in the present study. Therefore, further studies are needed to evaluate the clinical significance of Ki-67 in dogs with nasal sarcomas.

In this study, 68.4% (13/19) of nasal carcinomas were immunohistochemically positive for EGFR which correlates with the results of former studies (21, 22, 44). In human nasopharyngeal carcinomas, a correlation between EGFR expression and an advanced tumor stage has been demonstrated (67, 68). However, in the present study, there was no significant difference in EGFR expression among the 18 dogs with carcinomas classified into T-categories by cross-sectional imaging, which is similar to a former study by Fu (44). Therefore, no relation to larger tumor volumes or to an advanced stage of the disease can be suggested. Regarding correlation between EGFR expression and survival, in human undifferentiated nasopharyngeal carcinomas, a correlation between increased EGFR expression and shorter survival times was found (67, 68). Fu confirmed this observation in a study with dogs with nasal carcinomas including EGFR-negative carcinomas (44). Dogs with nasal carcinomas with higher EGFR expression had a significantly shorter MST after radiation therapy.

Due to the high number of EGFR-positive nasal carcinomas, TKI or monoclonal antibody targeting EGFR may represent a promising treatment strategy in dogs with nasal carcinomas (21, 22). Currently, several drugs targeting EGFR are available for humans, but their usability in veterinary medicine has not been investigated (69, 70). In clinical trials in humans with nasopharyngeal carcinoma, the EGFR-TKI cetuximab in combination with chemotherapeutic agents (carboplatin, cisplatin) and/or radiotherapy resulted in comparably favorable survival times with controllable side effects (71–75). The EGFR-TKI icotinib also has a radiosensitising effect in preclinical studies on cell lines of human nasopharyngeal carcinomas (68) and was well tolerated clinically in a phase I study with concurrent intensity-modulated radiotherapy (76).

While canine mammary tumors have been comparatively frequently immunohistochemically investigated for E-cadherin expression, there is no publication yet on E-cadherin expression in canine malignant nasal tumors. As E-cadherin is only expressed in epithelial cells, nasal sarcomas (7/7) were E-cadherin negative and 100% of carcinomas (19/19) were E-cadherin positive. Regarding correlation between E-cadherin expression and tumor stages, different results are reported. Therefore, a lower E-cadherin expression at higher tumor stages has been demonstrated in human nasopharyngeal tumors in two studies (77, 78) and in contrast not in another study (79). In the present study, a tendency of E-cadherin expression being slightly lower in higher T-categories has been observed but was not statistically significant. Lower expression in higher T-categories would support the basic assumption that tumors in the higher T-categories might grow more aggressively, since more aggressive and invasively growing tumors often show lower E-cadherin expression (77, 78). On the other hand, the classification of a tumor into a T-category is not only based on invasiveness, but also on tumor size at the time of diagnosis and thus may simply be more advanced.

Regarding dogs characteristics, comparable to other studies, dogs in this investigation showed a high average age of 10 years and an almost equal gender distribution (54% female, 46% male) (1, 3, 80, 81). Additionally, the long duration of clinical signs until diagnostics with on average 3 months was comparable to other studies, as, e.g., reported by Madewell et al. (mean 3.81 months) (1), Rassnick et al. (median 2 months) (82) and Meler et al. (mean 6.3 months) (7). This could be due to the fact that many owners underestimate nasal discharge in dogs or that clinical signs are initially alleviated by therapy with antibiotics, NSAIDs or corticosteroids (6). In this study, there was a non-significant difference in duration of clinical signs before diagnosis in dogs with carcinomas and sarcomas, with a shorter period of clinical signs in sarcomas than in carcinomas. This could be related to the fact that swelling in the tumor area and epistaxis are more common observed in dogs with non-epithelial tumors (3) and these clinical signs are maybe assessed as more serious by the owners leading to a sooner presentation to their regular veterinarian.

Regarding categorization into T-categories it has to be mentioned, that 24 of 26 dogs (92%) were examined by CT and 1 of 26 dogs (4%) by MRI. The use of MRI images for the diagnosis of a nasal tumor in dogs is just as suitable as the use of CT images (83, 84). However, when classifying T-categories according to Adams et al. (18), it should be noted that tumor extension may be estimated somewhat greater in MRI images than in CT images (85). As mentioned above, receptor expression was not significantly different according T-categories.

## Conclusion

The expression of VEGFR-2 and COX-2 was comparable between carcinomas and sarcomas and EGFR was detected in the majority of nasal carcinomas. Thus, an adjuvant or additional therapy with TKIs and/or selective COX-2 inhibitors could be a promising therapeutic strategy for both tumor types and should be investigated in further, prospective studies.

## Data availability statement

The original contributions presented in the study are included in the article/supplementary material, further inquiries can be directed to the corresponding author.

## Ethics statement

The animal study was approved by Regional Council of the Free State of Saxony, Leipzig, Germany: TVV Animal experiment subject 02/18. The study was conducted in accordance with the local legislation and institutional requirements.

## Author contributions

LP: Data curation, Formal analysis, Investigation, Methodology, Software, Validation, Visualization, Writing – original draft, Writing – review & editing. JJ: Data curation, Formal analysis, Methodology, Project administration, Supervision, Writing – original draft, Writing – review & editing. GO: Conceptualization, Data curation, Formal analysis, Funding acquisition, Investigation, Methodology, Project administration, Resources, Supervision, Validation, Writing – original draft, Writing – review & editing. MH-T: Conceptualization, Formal analysis, Funding acquisition, Investigation, Methodology, Project administration, Resources, Supervision, Validation, Writing – original draft, Writing – review & editing, Data curation. SR: Conceptualization, Data curation, Formal analysis, Funding acquisition, Investigation, Methodology, Project administration, Resources, Software, Supervision, Validation, Visualization, Writing – original draft, Writing – review & editing.

## Footnotes

^1^Fiji Image J, Fiji contributors, https://imagej.net/
